# Predictors of Meat Reduction: The Case of Slovenia

**DOI:** 10.3390/foods13152346

**Published:** 2024-07-25

**Authors:** Andrej Kirbiš, Vanesa Korže, Maruša Lubej

**Affiliations:** Department of Sociology, Faculty of Arts, University of Maribor, 2000 Maribor, Slovenia

**Keywords:** meat reduction, meat consumption, health, food, diet

## Abstract

Health, environmental, and animal advocacy organisations emphasise reducing or eliminating high meat consumption due to its adverse effects on health, sustainability, climate change, and animal welfare. Increasingly, people are deciding to reduce their meat consumption frequency. Our study aimed to examine predictors of meat reduction among Slovenian consumers, focusing on gender, age, partner and children status, size of residential settlement, socioeconomic status, and political orientation. We conducted a survey using non-probability sampling. We examined demographic, socioeconomic, and political predictors of individuals’ self-assessed intent to reduce meat consumption in the month following the survey. Additionally, we analysed respondents’ meat reduction during the three years prior. A correlation analysis revealed that higher age and education levels were significantly positively correlated with meat reduction patterns. An ordinal logistic regression analysis indicated that age was the only significant predictor of meat reduction intentions and past behaviour. Our findings suggest that middle-aged and elderly individuals are more likely than younger adults to report meat reduction behaviours. Future public health interventions should tailor approaches to different age groups, and, in particular, target younger individuals. Educational campaigns should highlight the health and environmental benefits of reducing meat and animal product consumption, particularly in primary and secondary schools.

## 1. Introduction

Health, environmental, and animal advocacy organizations emphasise the importance of reducing or eliminating high meat consumption due to its adverse effects on health, sustainability, climate change, and animal welfare [1,2,3,4]. The World Health Organization has classified red meat as likely to be carcinogenic to humans and processed meat products as carcinogenic [5,6]. Moreover, studies have linked (red) meat consumption to an increased risk of developing cancer [7,8,9], cardiovascular disease [10,11,12] and type 2 diabetes [13,14,15]. Furthermore, the production of animal-based foods produces twice as much global greenhouse gas emissions as plant-based foods [16] and livestock takes up 77% of global farming land [17].

Consequently, there is an increasing public awareness about the negative effects of excessive meat consumption. Increasingly, more people are deciding to reduce their meat consumption frequency or adopt a more plant-based diet. Devoted meat reducers are sometimes also categorized as partial vegetarians or flexitarians [18,19].

Several studies have examined meat consumption patterns among Slovenians. In a sample of the Slovenian population, 1.8% of adults and 2.6% of young Slovenians reported not eating red meat but consuming fish, poultry, milk products, or eggs. Additionally, 0.2% of adults self-classified as lacto-ovo vegetarians and 0.5% as vegans [20]. In another study among Slovenian adults and elders, 1.6% of adults and 1% of elders reported not eating red meat and poultry. In addition, 8.8% of adults and 8.9% of elders do not eat fish or fish products [21]. Kirbiš and colleagues [22] reported that 1.4% of Slovenians never eat meat and 3.9% never eat fish, while 6.4% consume meat occasionally or even less frequently. Kamin and colleagues [23] found that approximately 79% of young Slovenian meat reducers describe themselves as omnivores and 1.9% as flexitarians. They eat meat only occasionally, or less frequently than the average Slovenian.

### Study Aim

Despite the growing literature, scholars have argued that there is still a lack of systematic empirical studies about individuals who have decided to reduce their meat consumption [18,24,25]. In addition, most existing studies about meat reducers/flexitarians have been conducted in English-speaking countries [26,27,28] and Western European countries [19,29,30].

The current study aims to fill this gap by examining predictors of meat reduction in a post-socialist country. This geographical area remains significantly understudied. In fact, no study has yet explored the determinants influencing the reduction of meat consumption among adults from this European region. Existing studies of public dietary patterns from East and Central Europe, for example, show rather unhealthy and unsustainable dietary intake. These are combined with traditional and non-tolerant attitudes toward sustainable food choices and dietary minorities (e.g., vegans and vegetarians) [22].

Our study is based on a sample from Slovenia, one of the post-socialist Southeastern European countries. While there are some Slovenian studies on flexitarians and their motives [23,31], the samples included young people only. Moreover, Slovenian studies examined different motives for meat reduction, such as its impact on the environment, physical appearance and animal welfare, but did not specifically analyze demographic, socioeconomic and political predictors of meat reduction. In addition, existing studies from Western countries provide several inconsistent findings, for example, whether lower age and higher income increase or decrease meat reduction.

## 2. Theoretical Background

### 2.1. Sociodemographic Factors and Meat Reduction

#### 2.1.1. Gender

There are various predictors of the reduction of meat consumption patterns. Studies show that women more often than men decide to reduce their meat consumption or plan to do so in the future [24,32,33,34]. For example, Hielkema and Lund [30] reported that in Denmark, men were less inclined to reduce meat consumption and less likely to be vegetarian/vegan. Similarly, Malek and Umberger [25] found that in a sample of Australians (52.9% women), 63.2% of identified meat reducers were women. Men may be less willing to reduce meat consumption due to traditional gender norms whereby in different cultures eating meat is equated with masculinity [35].

#### 2.1.2. Age

Compared to studies on gender differences, previous studies give mixed results about the relationship between individuals’ age and willingness to reduce meat consumption. Several existing studies show that younger people represent the largest share of vegetarians/vegans [36,37,38] and that younger individuals are more willing to reduce meat consumption and follow a more plant-based diet [30,33,39]. On the other hand, some studies suggest that older people are more likely to reduce the frequency of meat consumption [27,40].

#### 2.1.3. Urban Environment

Existing research consistently shows that a higher proportion of meat reducers live in urban environments than in rural areas [19,38,41,42]. Urban-rural differences have been reported, for example, in Denmark [30], the Netherlands [19,42] and in Australia [43]. There are several mechanisms that may explain urban-rural differences. For example, residents of urban environments have higher levels of education than those from rural environments and a higher educational level increases the willingness to reduce meat consumption [32].

#### 2.1.4. Partner Status and Children in the Household

Studies also show that household characteristics, including partner and child status, impact the likelihood of meat reduction among individuals. Generally, meat eaters seem more likely to live with their partner, compared to meat reducers. In a sample from Australia, more unrestricted omnivores than meat reducers lived with their partner [25]. Similarly, in a study from Great Britain, a somewhat higher proportion of meat eaters lived only with their partner and without children, compared to meat reducers [40].

In addition, individuals with children in the household are more often omnivores and are less willing to reduce their meat consumption. For example, a higher share of avid meat eaters lived with their partner and children, compared to committed meat reducers [19]. Verain and colleagues [44] reported that compulsive meat eaters were somewhat more likely to live with a partner and kids, compared to meat reducers. However, meat lovers were least likely to live with a partner and kids (ibid.).

### 2.2. Socioeconomic Factors and Meat Reduction

#### 2.2.1. Educational Level

Studies almost uniformly show that individuals with a higher educational level are more likely to reduce the frequency of meat consumption [32,45,46]. For example, Verain and Dagevos [19] found that in The Netherlands, 44% of dedicated meat reducers had a high level of education and 19.3% had a low level of education, compared to avid meat eaters of whom 32% had a high level of education and 21.2% had a low level of education. Kirbiš and colleagues [22] reported that in Slovenia, a higher level of education was an independent predictor of less frequent meat consumption. Individuals with a greater amount of educational, financial, and other resources usually have a higher level of culinary knowledge, are more open to new types of food and are more familiar with different types of products and world cuisines [47].

#### 2.2.2. Income

Existing studies give mixed findings regarding the relationship between income level and reduction of meat consumption. Some studies show that individuals with a lower income are more likely to decide to reduce their meat consumption [27]. However, some studies [39,43] show individuals with higher incomes are more likely to reduce meat consumption.

### 2.3. Political Orientation and Meat Reduction

Finally, existing studies show there is a consensus that left or liberal-oriented individuals are more willing to reduce the frequency of meat consumption, compared to rightists or conservatives [30,48,49,50]. Furthermore, vegetarianism and veganism are usually perceived as a characteristic of the political left [51]. Right-wing individuals are more likely to advocate that humans have superiority over animals and maintain the carnist status quo, making them less likely to reduce meat consumption [52].

### 2.4. Study Hypotheses

Based on the findings of previous findings, we examined several potential predictors of meat reduction patterns among Slovenian adults. We were interested in the proportion of the sample indicating past and future meat reduction and in the effects of demographic, socioeconomic, and political determinants on meat reduction. We hypothesized that females (H1), younger people (H2), urban residents (H3), people without children (H4), those without partners (H5), individuals with higher education levels (H6), those with higher incomes (H7), and those with a leftist political orientation (H8) would be significantly more likely to report reducing meat consumption. We used data from a survey conducted in 2020 and applied logistic regression models to test our hypotheses.

## 3. Methodology

### 3.1. Sample Description

In May and June 2020, a cross-sectional survey was conducted. Utilising the snowball sampling method, adults aged 18 and over were invited to participate in a non-probability sample survey through the University of Maribor and various Slovenian social media platforms (Facebook, Twitter, and Instagram) as well as through emails. Participants were also asked to share the survey link among their acquaintances and peers in schools and workplaces. Data collection was facilitated using the online survey tool called “1ka.si”. The primary criteria for inclusion were being over 18 years of age and residing in Slovenia. Participants were required to read a written consent form and explicitly agree to participate in the study and consent to the publication of the results. Following consent, respondents were asked to complete a survey that gathered information on their dietary patterns, attitudes and behaviour, along with their sociodemographic and socioeconomic details.

The sample consisted of 390 respondents from Slovenia (81.79% female and 18.21% male). Participants were between 17 and 80 years old (M = 41.84, SD = 15.68). Most respondents lived in one of the two largest Slovenian cities (Ljubljana or Maribor) (38.21%), followed by those living in rural settlements (20.51%), small towns (20.00%), medium-sized cities (11.28%) and small cities (10.00%). Most participants in the sample were highly educated; 26.41% had a higher education degree or completed professional higher education, 30.77% held a university or master’s degree, 18.46% completed gymnasium, 13.59% had secondary technical education, 3.59% had lower or middle vocational education, and 1.79% reported having a PhD. The financial situation of the participants was self-assessed on a 1–10 scale with a mean score of 5.80 (SD = 1.47). Additionally, the average political orientation score was 4.75 (SD = 2.26), indicating a left-leaning tendency in political views among the participants.

### 3.2. Measures

#### 3.2.1. Anticipated Change in Meat Consumption

The extent of the anticipated change in meat consumption over the next month was assessed using a single item: “Please think about the next month. Do you think that the frequency of meat consumption (including red meat, processed meat, poultry, fish and seafood) will change for you personally over the next month? Thank you for your honest answer.” The response options were: “I will not eat meat” (1), “I will exclude meat from my diet” (2), “I will significantly reduce the amount of meat in my diet” (3), “I will slightly reduce the amount of meat in my diet” (4), “I will maintain approximately the same amount of meat” (5), “I will slightly increase the amount of meat in my diet” (6), and “I will significantly increase the amount of meat in my diet” (7). The first two groups were combined due to the low percentage of answers “I will exclude meat from my diet” (0.3%). The largest group (37.95%) indicated they would maintain the same level of meat consumption.

#### 3.2.2. Reported Change in Meat Consumption

The reported change in meat consumption over the past three years was measured with the item: “Now we would like to know whether the frequency of meat consumption (including red meat, processed meat, poultry, fish and seafood) has changed for you personally over the last three years? Thank you for your honest answer!” Response options included: “I haven’t eaten meat” (1), “I have excluded meat” (2), “I have significantly reduced the amount of meat in my diet” (3), “I have slightly reduced the amount of meat in my diet” (4), “I have maintained approximately the same amount of meat consumption” (5), “I have slightly increased the amount of meat in my diet” (6), and “I have significantly increased the amount of meat in my diet” (7). Similarly to the anticipated change in meat consumption, we combined the first two groups in the reported change in meat consumption. Most respondents (36.68%) reported maintaining the same level of meat consumption in the three years prior to the survey.

#### 3.2.3. Predictors

Gender was recorded as a binary variable with two categories: male (1) and female (2). The sample was predominantly female (81.79%). Age was measured in years. The mean age of respondents was 41.84 years (SD = 15.68).

Size of place of residence was categorized into five groups: village/rural settlement (under 1000 inhabitants) (1), small town (1000–5000 inhabitants) (2), small city (5000–10,000 inhabitants) (3), medium-sized city (10,000–100,000 inhabitants) (4), and Ljubljana or Maribor (the largest cities in Slovenia with over 100,000 inhabitants) (5). Most of the participants lived in the two large cities (38.21%).

Educational level was assessed with a single item of eight categories: (1) primary school or less; (2) lower or middle vocational education (2–3 years); (3) secondary technical education (4 years); (4) gymnasium; (5) higher education, professional higher education or university programme at the first Bologna level (level VI/1 and VI/2); (6) bachelor’s degree, second Bologna cycle master’s degree, specialisation in higher professional education, (7) master of science; specialisation in the pre-degree university programme (level VIII/1); and (8) doctor of science (level VIII/2). Most respondents completed higher education, professional higher education or university programmes at the first Bologna level (26.41%).

Participants rated their financial situation on a scale from 1 to 10, with 1 indicating a below-average financial situation and 10 indicating a high financial situation. The mean score was 5.80 (SD = 1.47).

Living with a partner was assessed with a single item, indicating whether the respondent lived without (0) or with a partner (1). Most respondents reported living with a partner (61%).

Living with a child was also assessed with a single item, indicating whether the respondent lived without (0) or with (1) a child. Most respondents reported living without a child (66.2%).

Political orientation was measured on a scale from 0 to 10, with 0 indicating the most left-leaning orientation and 10 indicating the most right-leaning orientation. The mean score was 4.75 (SD = 2.26).

### 3.3. Analytical Strategy

We created two models to test our hypotheses. In the first model, we explored the predictors of future meat consumption intentions on the entire sample, which consists of omnivores, vegans, and vegetarians, since we could not eliminate the possibility that the last two groups might plan on changing their eating patterns (i.e., start eating meat) in the future. In the second model, we explored predictors of past meat consumption patterns. In that model, we used only the subsample of our data, in which only the omnivores were included (*n* = 319).

For both models, ordinal logistic regression analysis was used due to the ordinal nature of our outcome variables. Additionally, in two of the variables that were used for the analysis, there was a notable percentage of missing entries. In the sample as a whole (Model 1), 27.44% of answers related to the political orientation variable were missing, and 37.69% of answers related to the future eating intentions variable were missing. In addition, 27.44% of answers related to the political orientation variable were missing in our subsample for Model 2.

We assessed the missing data mechanism using Little’s MCAR test, which indicated a significant result (first model: χ^2^(23) = 77.4, *p* < 0.001; second model: χ^2^(16) = 56.7, *p* < 0.001), thus rejecting the assumption of missing completely at random (MCAR). However, binomial logistic regression analyses on the missingness indicators confirmed that the data were missing at random (MAR). Therefore, we followed the procedure proposed by van Klingeren and de Graaf [53] and conducted multiple imputations using the MissRanger package in R (version 2024.04.0+735). This procedure is a robust method for handling missing data that uses predictive mean matching and random forests. We performed multiple imputations with 20 iterations to ensure stable estimates. Rubin’s Rules were used to pool the estimates from multiple imputed datasets.

Sensitivity analysis was conducted by varying imputation settings. This demonstrated that our conclusions were robust. Specifically, we used alternative settings with 50 trees and three predictive mean matching (PMM) neighbours to confirm robustness. Furthermore, we utilized bootstrapping with 1000 resamples to examine the variability and stability of the coefficients in both models. All variance inflation factors (VIF) were well below the commonly accepted threshold of 5, indicating that multicollinearity was not a concern in our models.

Model fit was evaluated based on the AIC and null deviance, with the values for the first model being 808.9739 and 784.9739, respectively. These metrics, alongside the mentioned satisfactory test for the proportional odds assumption in that model (Omnibus χ^2^(24) = 21.57, *p* = 0.60), confirmed the adequacy of the model. Similarly, the second model’s fit was adequate, with an AIC of 944.6312 and null deviance of 918.6312. The proportional odds assumption of the second model was met as well (Omnibus χ^2^(32) = 38.96, *p* = 0.19).

The analyses in the results section present unstandardized coefficients (β) and their significance levels. Adjusted proportional odds ratios (AOR) are provided for each significant predictor. Statistical significance was set at *p* < 0.05.

## 4. Results

### 4.1. Descriptive Analysis

Model 1 included 390 respondents, and Model 2 included 319 respondents. Descriptive statistics are presented in Table 1. Females were overrepresented in both samples (81.79% in Model 1 and 79.94% in Model 2). The average age for both samples was 42 years, and a notable portion of the respondents reported living in larger Slovenian cities (Ljubljana or Maribor), followed by inhabitants of rural settlements and small towns. Most respondents were highly educated, with the majority holding higher education degrees and over half (61% in Model 1 and 61.13% in Model 2) reported living with a partner, while only a third indicated they live with a child (33.8% in Model 1 and 34.80% in Model 2). The financial situation of respondents was around the nominal middle value, with mean scores around 5.8 on a 1–10 scale. The predominant political orientation was left-leaning.

The largest group in the sample included in Model 1 intended to maintain their meat consumption (37.95%) over the next month, while a significant portion of the sample in Model 2 (consisting of omnivores only) reported having approximately the same meat consumption over the past three years (36.68%).

### 4.2. Bivariate Analysis

The correlation analysis (Table 2) showed that age was significantly negatively correlated with meat consumption intentions (r = −0.18, *p* = 0.004) and with past meat consumption (r = −0.18, *p* = 0.002) (Table 3), indicating that older respondents were more likely to decrease meat consumption in the following month. They were also more likely to have reduced their meat consumption in the past three years. Educational level was also significantly negatively correlated with past meat consumption patterns (r = −0.12, *p* = 0.03), with more educated more likely to report a decrease in meat consumption, while none of the other tested predictors were significantly correlated with either future intentions or past meat consumption.

### 4.3. Multivariate Analysis

In our multivariate analysis, which used ordinal logistic regression (Table 4 and Table 5), age was again the only significant predictor of meat consumption intentions (AOR = 0.98, *p* = 0.002) and of past meat consumption patterns (AOR = 0.98, *p* = 0.002), with older individuals showing higher odds of intending to decrease meat consumption in the following month and higher odds of having decreased meat consumption in the past three years. Specifically, for each additional year of age, the odds of decreasing meat consumption is increased by 2%. Gender, living in an urban environment, higher education, higher income, living with children or a partner, and political orientation were not significant predictors of meat reduction in either sample.

For both models shown in Table 4 and Table 5, the Brant Test showed that the *p*-values were greater than 0.05 (Model 1: *p* = 0.60; Model 2: *p* = 0.19), indicating that the proportional odds assumption holds, which justifies the use of ordinal logistic regression. The model fit for Model 1 (AIC = 808.97) and Model 2 (AIC of 944.63) was adequate. As indicated with Nagelkerke R^2^, Model 2 had slightly higher explanatory power (R^2^ = 0.05) compared to Model 1 (R^2^ = 0.06), although both were low. A detailed model fit statistic table, including null and residual deviance and Cox & Snell R^2^, can be found in Appendix A.

Finally, we performed bootstrapping (with 1000 resampling with replacement) to test the robustness of our results. Table 6 shows the bootstrap confidence interval (CI) values for both models. The confidence intervals for the variable age do not include the value of zero, confirming increased age as a significant predictor of meat reduction intentions and past meat reduction.

Overall, the results show that higher age was a consistent predictor of past and intended meat reduction patterns, while other demographic and socioeconomic variables did not have a significant impact on either of the two outcome variables.

## 5. Discussion

Our study examined demographic, socioeconomic, and political predictors of meat reduction among Slovenians. Slovenia presents a unique case, as previous studies have shown a public aversion to sustainable food practices and attitudes [22]. Unfavourable public attitudes towards sustainable diets are also reflected among various professional groups, notably within the medical field. For example, the Slovenian Prime Minister established the Strategic Council for Nutrition (SCN) to gather specialists from diverse sectors not typically involved in government decision-making [54]. In 2023, the SCN discussed recommendations and guidelines to introduce plant-based menus in kindergartens and primary schools. This initiative aimed to provide an additional choice for parents desiring a more sustainable diet for their children, in line with the latest dietary recommendations [54]. However, groups of Slovenian professionals, including paediatricians, opposed these recommendations [55], disregarding established medical and health institutions’ endorsements of plant-based diets [56,57,58]. Additionally, other Slovenian medical and public health professionals and institutions promote various animal-based products as “healthy” and “nutritious” [59,60,61]. Similarly, the Slovenian Ministry of Agriculture, Forestry, and Food implements the “EU school scheme” [62], which “supports the distribution of milk, fruit and vegetables to millions of children, from nursery to secondary school, across the EU” [63]. This highlights the significant resistance among both the general public and professionals in Slovenia to adopting more sustainable dietary practices.

In our multivariate analysis, age was the only significant predictor of both past meat consumption patterns and future meat consumption intentions, with older individuals more likely to have reduced their meat intake and intend to do so in the future. Gender, urban living environment, educational level, income, living with a partner or children, and political orientation had no significant impact.

Our findings regarding age align with those of Neff and colleagues [27] and Apostolidis and McLeay [40], who also found that older individuals were more likely to reduce meat consumption. Previous research suggests that older meat reducers cite health awareness, the higher price of meat [27,40] and a decreased appetite for meat [64], as their main reasons for reducing meat consumption. Older Slovenians may also reduce their meat intake for similar reasons, although future studies are needed to examine the motives behind their dietary preferences and health considerations.

Contrary to numerous studies that found women are more likely to reduce meat consumption [24,25,32], our results found no gender effect. This may indicate that the typically found association of meat consumption with masculinity [35] might not be as pronounced in Slovenia, or that gender differences in gender role attitudes in Slovenia may be smaller than in Western societies. However, as our sample had an overrepresentation of women, these results should be interpreted cautiously.

While earlier studies point towards a link between settlement size and reduced meat consumption [19,38,41,42], we did not detect such a link in Slovenia. That could be because urban areas in Slovenia are relatively small and culturally more homogeneous compared to urban environments in other countries [65], which could reduce the urban-rural divide in cultural influences on meat consumption behaviours. Cultural homogeneity in Slovenia might be reflected in the relatively consistent dietary patterns observed across different settlement sizes within our sample.

Additionally, higher educational levels [32,45,46], income levels [39,43], and a leftist political orientation [30,48,49], which were previously found to be linked to reduced meat consumption, were non-significant in our multivariate models. This could partly be explained by the limited variability in those variables within our sample, for instance, the financial situation assessment averaged around 5.80 on a scale of 1–10, with a low standard deviation of 1.45–1.47. Similarly, the distribution of educational levels showed a relatively even spread, with most participants having higher education or university degrees. The limited variability of Slovenian social structure reflects Slovenia’s relatively small levels of inequality in income [66] and education [67]. One of the reasons for comparatively small inequalities in various country characteristics may be a consequence of the past, as Slovenia and other former socialist countries in the Southeast and Central European region have a history of egalitarian social policies and public’s cultural orientations that may reduce the differences in lifestyles between demographic, social, cultural, and economic groups, including in dietary patterns.

The findings of this study have important policy implications. For example, given the non-significant predictors, broad-based strategies that address the whole population rather than focusing on specific demographics (with the exception of age) or socioeconomic groups may be more effective. Policymakers should also consider initiatives that increase awareness of the environmental and ethical benefits of reducing meat intake to particularly target younger people who might be more responsive to messages about environmental concerns and animal welfare, as indicated in previous research [23,42,64]. Educational campaigns should highlight the benefits of reducing meat and animal product consumption, particularly in primary and secondary schools. In addition, our results suggest that determinants of dietary patterns in post-socialist countries may differ from Western, high-income countries, which indicates the need for further studies of social and economic determinants of dietary patterns in non-Western countries.

Our study has several limitations. First, it is cross-sectional and, therefore, it did not allow us to separate between cause and effect. Second, our sample was overrepresented by females (81.79% and 79.94% in Models 1 and 2, respectively) and urban residents. That may limit the generalizability of our results to the broader Slovenian and European population. Future research would benefit from more balanced gender representation to further validate these findings. Third, despite using ordinal logistic regression models appropriate for our outcome variables, the low explanatory power indicated by Nagelkerke R^2^ values in both models suggests that other factors that were not included in our models may influence meat consumption patterns. Lastly, although we addressed the notable percentage of missing data in two variables through multiple imputations and confirmed the robustness of our conclusions via sensitivity analysis and bootstrapping, the high proportion of missing data may have nonetheless partly impacted the reliability of our findings.

Future research should aim to include representative or more gender-balanced samples and consider additional possible predictors of meat reduction behaviour and intentions. The reasons behind higher meat reduction intentions among older people in Slovenia should be explored further. Additionally, future research could explore other potential predictors, such as cultural characteristics and psychological determinants, which may provide a better understanding of meat reduction behaviours in Slovenia and similar post-socialist contexts.

## 6. Conclusions

We found that age is a significant predictor of meat reduction behaviour among Slovenian adults, with middle-aged and older individuals more likely to reduce their meat intake. However, other demographic, socioeconomic, and political factors do not significantly influence meat reduction patterns in the post-communist country of Slovenia. Our findings suggest that public health strategies should consider targeting the general population to reduce meat consumption among Slovenians. Additionally, initiatives that increase awareness of the environmental and ethical aspects of meat consumption and production should be emphasized, since they resonate more with the younger population. This is especially critical as the younger population’s lower likelihood of meat reduction patterns will have a negative effect on the environment and climate change in the future. Educational initiatives targeting the negative health, environmental and ethical implications of meat eating may have a beneficial effect on the public’s awareness of the role of food items on their plates. Finally, further research in post-communist cultural environments is needed to explore other potential predictors and to confirm these findings in representative national samples and longitudinal studies.

## Figures and Tables

**Table 1:** Descriptive analysis.

	Sample for Model 1 (*n* = 390)			Sample for Model 2 (*n* = 319)		
Variables	Mean (SD)	Percentage	Missing (%)	Mean (SD)	Percentage	Missing (%)
Anticipated change in the frequency of meat consumption over the next month			37.69%			
I will not eat meat/I will exclude meat from my diet.		5.90%				
I will significantly reduce the amount of meat in my diet.		5.64%				
I will slightly reduce the amount of meat in my diet.		12.05%				
I will maintain approximately the same amount of meat.		37.95%				
I will slightly increase the amount of meat in my diet.		0.77%				
I will significantly increase the amount of meat in my diet.		0%				
Reported change in the frequency of meat consumption over the last three years			0%			
I haven’t eaten meat/I have excluded meat.					9.72%	
I have significantly reduced the amount of meat in my diet.					23.51%	
I have slightly reduced the amount of meat in my diet.					24.76%	
I have maintained approximately the same amount of meat consumption.					36.68%	
I have slightly increased the amount of meat in my diet					3.45%	
I have significantly increased the amount of meat in my diet.					1.88%	
Gender			0%			0%
Male		18.21%			20.06%	
Female		81.79%			79.94%	
Age (years)	41.84 (15.68)		0.26%	42.04 (15.65)		0.31
Size of place of residence						0%
Village/rural settlement (under 1000 inhabitants)		20.51%			21.00%	
Small town (1000–5000 inhabitants)		20.00%			21.32%	
Small city (5000–10,000 inhabitants)		10.00%			10.34%	
Medium-sized city (10,000–100,000 inhabitants)		11.28%			10.97%	
Ljubljana or Maribor		38.21%			36.36%	
Level of education			0%			0%
Primary school or less (1st and 2nd level)		0%			0%	
Lower or middle vocational education (2–3 years) (3rd and 4th level)		3.59%			4.39%	
Secondary technical education (4 years) (5th level)		13.59%			15.05%	
Gymnasium (5th level)		18.46%			16.93%	
Higher education, professional higher education, or university programme of the 1st Bologna cycle (6/1 and 6/2 level)		26.41%			25.71%	
University programme; university master’s programme of the 2nd Bologna cycle; specialization after professional higher education programme (7/1 level)		30.77%			31.35%	
Master’s degree in science; specialisation after pre-Bologna university programme (7/2 level)		5.38%			4.70%	
Doctorate in science (8/2 level)		1.79%			1.88%	
Financial situation assessment (scale 1–10) 1 = below average; 10 = high	5.80 (1.47)		0%	5.86 (1.45)		0%
Living with a partner (Q2a)			0%			0%
No		39%			38.87%	
Yes		61%			61.13%	
Living with a child (Q2b)			0%			0%
No		66.2%			65.20%	
Yes		33.8%			34.80%	
Political orientation (scale 0–10) 0 = left; 10 = right	4.75 (2.26)		27.44%	4.81 (2.22)		23.82%

**Table 2:** Spearman correlation mtrix with *p*-values for Model 1.

Variable	Future Meat Eating Intentions	Gender	Age	Settlement Size	Level of Education	Financial Situation	Living with/without Partner	Living with/without a Child	Political Orientation
Future meat-eating intentions	1.00	−0.01 (0.99)	−0.18 ** (0.004)	−0.09 (0.17)	−0.05 (0.42)	−0.05 (0.42)	0.03 (0.62)	0.03 (0.66)	−0.03 (0.68)
Gender	−0.01 (0.99)	1.00	−0.13 * (0.01)	−0.12 * (0.015)	0.02 (0.66)	0.01 (0.82)	0.07 (0.16)	0.03 (0.53)	0.08 (0.16)
Age	−0.18 ** (0.004)	−0.13 * (0.01)	1.00	0.17 ** (0.0005)	0.11 * (0.04)	−0.08 (0.09)	0.28 ** (<0.001)	0.27 ** (<0.001)	−0.10 (0.10)
Settlement size	−0.09 (0.17)	−0.12 * (0.015)	0.17 ** (0.0005)	1.00	0.19 ** (0.0001)	0.03 (0.53)	0.04 (0.45)	−0.04 (0.46)	−0.14 * (0.02)
Level of education	−0.05 (0.42)	0.02 (0.66)	0.11 * (0.04)	0.19 ** (0.0001)	1.00	0.24 ** (<0.001)	0.13 * (0.01)	0.15 ** (0.004)	−0.05 (0.43)
Financial situation	−0.05 (0.42)	0.01 (0.82)	−0.08 (0.09)	0.03 (0.53)	0.24 ** (<0.001)	1.00	0.02 (0.73)	−0.02 (0.76)	0.00 (0.72)
Living with/without a partner	0.03 (0.62)	0.07 (0.16)	0.28 ** (<0.001)	0.04 (0.45)	0.13 * (0.01)	0.02 (0.73)	1.00	0.37 ** (<0.001)	−0.05 (0.39)
Living with/without a child	0.03 (0.66)	0.03 (0.53)	0.27 ** (<0.001)	−0.04 (0.46)	0.15 ** (0.004)	−0.02 (0.76)	0.37 ** (<0.001)	1.00	0.12 * (0.05)
Political orientation	−0.03 (0.68)		0.08 (0.16)	−0.14 * (0.02)	−0.05 (0.43)	0.00 (0.72)	−0.05 (0.39)	0.12 * (0.05)	1.00

Note. Values in parentheses indicate *p*-values. * *p* < 0.05, ** *p* < 0.01.

**Table 3:** Spearman correlation matrix with *p*-values for Model 2.

Variable	Past Meat Consumption	Gender	Age	Settlement Size	Level of Education	Financial Situation	Living with/without Partner	Living with/without a Child	Political Orientation
Past meat consumption	1.00	−0.07 (0.24)	−0.18 ** (0.001)	−0.04 (0.47)	−0.12 * (0.03)	−0.09 (0.11)	−0.08 (0.15)	−0.07 (0.22)	0.06 (0.32)
Gender	−0.07 (0.24)	1.00	−0.14 * (0.01)	−0.14 * (0.01)	0.01 (0.87)	0.01 (0.87)	0.05 (0.37)	0.05 (0.34)	0.10 (0.11)
Age	−0.18 ** (0.002)	−0.14 * (0.01)	1.00	0.19 ** (<0.001)	0.10 (0.09)	−0.11 * (0.04)	0.23 ** (<0.001)	0.26 ** (<0.001)	−0.12 * (0.05)
Settlement size	−0.04 (0.47)	−0.14 * (0.01)	0.19 ** (<0.001)	1.00	0.18 ** (0.001)	0.02 (0.77)	0.03 (0.53)	−0.05 (0.34)	−0.14 * (0.02)
Level of education	−0.12 * (0.03)	0.01 (0.87)	0.10 (0.09)	0.18 ** (0.001)	1.00	0.26 ** (<0.001)	0.16 ** (0.004)	0.16 ** (0.004)	−0.03 (0.67)
Financial situation	−0.09 (0.11)	0.01 (0.87)	−0.11 * (0.04)	0.02 (0.77)	0.26 ** (<0.001)	1.00	0.00 (0.93)	−0.06 (0.29)	−0.04 (0.58)
Living with/without a partner	−0.08 (0.15)	0.05 (0.37)	0.23 ** (<0.001)	0.03 (0.53)	0.16 ** (0.004)	0.00 (0.93)	1.00	0.35 ** (<0.001)	0.11 (0.10)
Living with/without a child	−0.07 (0.22)	0.05 (0.34)	0.26 ** (<0.001)	−0.05 (0.34)	0.16 ** (0.004)	−0.06 (0.29)	0.35 ** (<0.001)	1.00	−0.04 (0.49)
Political orientation	0.06 (0.32)	0.10 (0.11)	−0.12 * (0.05)	−0.14 * (0.02)	−0.03 (0.67)	−0.04 (0.58)	0.11 (0.10)	−0.04 (0.49)	1.00

Note. Values in parentheses indicate *p*-values. * *p* < 0.05, ** *p* < 0.01.

**Table 4:** Ordinal logistic regression results for future meat consumption intentions (Model 1).

Predictor	β	SE	*p*-Value	AOR (CI)
Gender	−0.101	0.270	0.71	0.90 (0.53, 1.53)
Age	−0.022 **	0.007	0.002	0.98 (0.97, 0.99)
Settlement size	−0.103	0.070	0.14	0.90 (0.78, 1.03)
Level of education	−0.071	0.084	0.40	0.93 (0.79, 1.10)
Financial situation	−0.041	0.074	0.58	0.96 (0.83, 1.11)
Living with/without a partner	0.270	0.235	0.25	1.31 (0.83, 2.07)
Living with/without a child	0.134	0.247	0.59	1.14 (0.70, 1.86)
Political orientation	−0.024	0.047	0.61	0.98 (0.89, 1.07)

Note. Values in parentheses indicate *p*-values. ** *p* < 0.01.

**Table 5:** Ordinal logistic regression results for past meat consumption patterns (Model 2).

Predictor	β	SE	*p*-Value	AOR
Gender	−0.412	0.256	0.12	0.66 (0.40, 1.10)
Age	−0.021 **	0.007	0.002	0.98 (0.97, 0.99)
Settlement size	−0.019	0.067	0.78	0.98 (0.85, 1.13)
Level of education	−0.094	0.082	0.25	0.91 (0.76, 1.10)
Financial situation	−0.136	0.076	0.07	0.87 (0.73, 1.05)
Living with/without a partner	−0.123	0.226	0.58	0.88 (0.57, 1.34)
Living with/without a child	−0.044	0.237	0.85	0.96 (0.60, 1.53)
Political orientation	0.041	0.048	0.39	1.04 (0.95, 1.15)

Note. Values in parentheses indicate *p*-values. ** *p* < 0.01.

**Table 6:** Logistic regression results with bootstrap confidence intervals.

Model 1: Future Meat Consumption								
Predictor	Original β	Bootstrap Bias	Lower CI (95%)	Upper CI (95%)	Original AOR	Bootstrap Bias	Lower CI (95%)	Upper CI (95%)
Gender	−0.101	−0.018	−0.569	0.471	0.904	0.983	0.566	1.601
Age	−0.022	−0.0001	−0.035	−0.008	0.978	0.999	0.966	0.992
Settlement size	−0.103	−0.005	−0.243	0.044	0.902	0.995	0.784	1.045
Level of education	−0.071	−0.006	−0.234	0.096	0.932	0.994	0.791	1.101
Financial situation	−0.041	0.0002	−0.181	0.104	0.960	1.000	0.835	1.110
Living with/without a partner	0.270	0.00001	−0.216	0.768	1.310	1.000	0.806	2.156
Living with/without a child	0.134	−0.003	−0.400	0.668	1.144	0.997	0.671	1.951
Political orientation	−0.024	−0.001	−0.113	0.079	0.976	0.999	0.893	1.082
Model 2: Past meat consumption								
Gender	−0.412	−0.010	−0.918	0.104	0.662	0.990	0.399	1.109
Age	−0.021	−0.001	−0.034	−0.007	0.979	0.999	0.967	0.994
Settlement size	−0.019	0.002	−0.163	0.122	0.982	1.002	0.849	1.130
Level of education	−0.094	0.001	−0.276	0.091	0.910	1.001	0.759	1.096
Financial situation	−0.136	−0.002	−0.294	0.044	0.873	0.998	0.745	1.045
Living with/without a partner	−0.123	−0.009	−0.563	0.357	0.884	0.992	0.570	1.429
Living with/without a child	−0.044	−0.002	−0.568	0.433	0.957	0.998	0.566	1.542
Political orientation	0.041	−0.002	−0.057	0.142	1.042	0.998	0.945	1.153

## Data Availability

The original contributions presented in the study are included in the article, further inquiries can be directed to the corresponding author.

## Appendix A

**Table A1:** Logistic Regression for Missing Data Indicators.

	Model 1				Model 2	
	Political Orientation (Missing)		Future Meat Eating Intentions (Missing)		Political Orientation (Missing)	
Predictor	β	SE	β	SE	β	SE
Intercept	−4.135 ***	0.593	−3.058 ***	0.512	−4.4628 ***	0.6855
Gender	1.107 **	0.360	1.154 ***	0.320	1.1611 **	0.4076
Age	0.033 ***	0.008	0.021 **	0.007	0.0371 ***	0.0093
Settlement size	0.229 **	0.077	0.216 **	0.070	0.2084 *	0.0897

Note. Values in parentheses indicate *p*-values. * *p* < 0.05, ** *p* < 0.01, *** *p* < 0.001.

**Table A2:** Sensitivity Analysis for Imputation Settings.

	Model 1		Model 2	
Setting	AIC	Significant Predictors (Beta)	AIC	Significant Predictors
Standard	808.97	Age (−0.02 **)	944.63	Age (−0.02 **)
Alternative (50 trees, 3 PMM)	699.58	Age (−0.02 **)	945.11	Age (−0.02 **)

Note. ** *p* < 0.01.

**Table A3:** Model Fit Statistics for Model 1 and Model 2.

	Model 1	Model 2
AIC	808.97	944.63
Null Deviance	784.97	918.63
Residual Deviance	784.97	918.63
Cox & Snell R^2^	0.0407	0.0609
Nagelkerke R^2^	0.0467	0.0643

**Table A4:** Brant Test for Proportional Odds Assumption.

	Model 1			Model 2		
Predictor	χ^2^	df	*p*-Value	χ^2^	df	*p*-Value
Gender	5.76	3	0.12	5.17	4	0.27
Age	1.35	3	0.72	5.30	4	0.26
Settlement size	0.78	3	0.85	6.90	4	0.14
Level of education	0.73	3	0.87	8.55	4	0.07
Financial situation	1.27	3	0.74	1.53	4	0.82
Living with/without a partner	7.02	3	0.07	7.93	4	0.09
Living with/without a child	3.27	3	0.35	2.24	4	0.69
Political orientation	0.14	3	0.99	5.67	4	0.23
Omnibus	21.57	24	0.60	38.96	32	0.19
